# Generation of Physical Map Contig-Specific Sequences Useful for Whole Genome Sequence Scaffolding

**DOI:** 10.1371/journal.pone.0078872

**Published:** 2013-10-24

**Authors:** Yanliang Jiang, Parichart Ninwichian, Shikai Liu, Jiaren Zhang, Huseyin Kucuktas, Fanyue Sun, Ludmilla Kaltenboeck, Luyang Sun, Lisui Bao, Zhanjiang Liu

**Affiliations:** The Fish Molecular Genetics and Biotechnology Laboratory, Aquatic Genomics Unit, School of Fisheries, Aquaculture and Aquatic Sciences and Program of Cell and Molecular Biosciences, Auburn University, Auburn, Alabama, United States of America; University of North Carolina at Charlotte, United States of America

## Abstract

Along with the rapid advances of the nextgen sequencing technologies, more and more species are added to the list of organisms whose whole genomes are sequenced. However, the assembled draft genome of many organisms consists of numerous small contigs, due to the short length of the reads generated by nextgen sequencing platforms. In order to improve the assembly and bring the genome contigs together, more genome resources are needed. In this study, we developed a strategy to generate a valuable genome resource, physical map contig-specific sequences, which are randomly distributed genome sequences in each physical contig. Two-dimensional tagging method was used to create specific tags for 1,824 physical contigs, in which the cost was dramatically reduced. A total of 94,111,841 100-bp reads and 315,277 assembled contigs are identified containing physical map contig-specific tags. The physical map contig-specific sequences along with the currently available BAC end sequences were then used to anchor the catfish draft genome contigs. A total of 156,457 genome contigs (~79% of whole genome sequencing assembly) were anchored and grouped into 1,824 pools, in which 16,680 unique genes were annotated. The physical map contig-specific sequences are valuable resources to link physical map, genetic linkage map and draft whole genome sequences, consequently have the capability to improve the whole genome sequences assembly and scaffolding, and improve the genome-wide comparative analysis as well. The strategy developed in this study could also be adopted in other species whose whole genome assembly is still facing a challenge.

## Introduction

With the advances of sequencing technologies, genomes of many species with biological or economic importance are currently under sequencing. With the exception of PacBio sequencing platform, several nextgen sequencing technologies such as 454 sequencing, Illumina sequencing, and SOLiD sequencing produce relatively short sequencing reads [1-3], making subsequent sequence assembly a great challenge. 

With most eukaryotic genomes, several factors further complicate whole genome sequence assembly: 1) the genome size is most often large at billion base pair range; 2) most eukaryotic genomes contain repetitive elements that are either in tandem repeats or dispersed in the genome; 3) eukaryotic genomes are loaded with long tracts of simple sequence repeats (microsatellites) that most often pose sequencing challenges because of frequent terminations at such sites [4]. All these result in segmented genome assembly. Typically, repetitive DNA sequences such as transposons and short tandem repeats that are interspersed in the genome shatter the *de novo* assembly because the sequencing reads are not long enough to include the entire repetitive sequence plus unique flanking sequences. As a consequence, assembly algorithms cannot uniquely assign sequences that arise from within the repeat, resulting in shortened sequence contigs; similarly, sequencing reactions terminate when encountering microsatellite repeats causing assembly breakage at the microsatellite sites even though microsatellite loci themselves are relatively short, most often within the 100 bp in size. Of course, with a large genome and short contigs, the consequence is large numbers of contigs. 

Such challenges become even more significant when dealing with teleost fish genomes because they went through an additional round of whole genome duplication (the 3R hypothesis) [5,6]. With some teleost fish such as common carp (*Cyprinus carpio*), Atlantic salmon and other salmonid fishes, the situation is even more complex because their genomes went through yet another round of whole genome duplication (the 4R hypothesis) [7,8]. The duplicated genes cause confusions on assembly because the assembly software cannot uniquely place the highly conserved gene sequences into its own contigs, but rather in many cases, sequence stacking was resulted, leading to mistakes in sequence assembly.

Several pilot studies on genome sequence assembly of fish species have been conducted [4,9,10] utilizing the high throughput sequencing data generated by next-generation sequencing platforms. In all cases, however, it was concluded that it is difficult to achieve a good level of genome sequence assembly when employ high throughput sequencing data alone, primarily due to the repetitive sequences within the genomes. Apparently, longer reads or paired end reads with larger insert size may help go through the repetitive region and improve the assembly [4]. However, generation of long and accurate sequences has always been a challenge because of the involved high cost. Therefore, other genome resources such as genetic linkage map, BAC-based physical map, BAC end sequences are needed to improve the whole genome sequence assembly, especially for scaffolding. Even with such genomic resources, assembly of complex genomes, particularly for scaffolding, requires additional genome resources. For instance, the best genome sequence assembly historically relied upon the availability of sequences generated by using the “clone-by-clone” sequencing strategy [11]. Such successes come from: 1) The clone-by-clone approach allow generation and the assembly of sequences to be divided into local assemblies within a clone, thereby reducing the complexity of the sequences and their assembly. This approach minimizes the presence of more than one interspersed repeat in each clone, thus a sequence that is repetitive from a genome-wide perspective may now be unique in a clone; 2) By using the minimal tiling path, the locally assembled sequences can be assembled into scaffolds based on physical maps. However, the historical clone-by-clone strategy is too expensive and too laborious with traditional sequencing. The aim of this study was to determine if physical map contig-specific sequences can be generated using the nextgen sequencing, and if such sequences can bring existing small genome sequence contigs into scaffolds corresponding to the physical map contigs.

Channel catfish is the predominant aquaculture species in the United States. The channel catfish genome is estimated to be 0.95 Gb in size ([12]; www.genomesize.com). It is highly AT-rich, with 60.7% A+T [13]. The catfish genome contains one main type of tandem repeats named Xba elements [14] and several types of dispersed repetitive elements, with the predominant dispersed repetitive elements being Tc1/mariner DNA transposons [15]. In addition, retrotransposons, LINE and SINE elements also exist in the catfish genome, with several SINE elements being well characterized such as the *Mermaid* and *Merman* SINE elements [13,16-18]. Short tandem repeats (microsatellites) are also highly abundant in the catfish genome with AC and AG being the most abundant types of microsatellites [19-21]. All these repeats within the catfish genome added more complexities to whole genome sequence assembly. 

A pilot study for the catfish genome assembly was conducted [4]. In that study, we found that assembly of Illumina sequences was not very effective even with a reasonable fraction of sequences generated from 454 sequencing. Using only Illumina sequences, an initial draft genome assembly confirmed our conclusion, i.e., sequence contigs break at the repetitive sequences, most often at the Tc1 transposons or microsatellite sequences. As a result, use of only Illumina sequences resulted in a relative large number of sequences of over 200,000 contigs (unpublished data). 

A number of catfish genomic sequences are currently available to assist whole genome sequence assembly. These resources include genetic linkage maps [22-24], physical maps [25,26], BAC end sequences (BES) [13,17], and integrated linkage and physical maps using BES-derived markers [23]. Use of these resources will definitely enhance whole genome sequence assembly. For instance, BES are useful to bring contigs into scaffolds because BAC end sequences from both ends are available [13,17] and they span an average distance of 161 kb [27]. The BAC-end sequences associated BAC clones can relate them to the physical map; the integrated physical and linkage map can bring the physical map contigs to linkage groups (chromosomes). However, as the anchor point to link the genome contigs, the BAC end sequences are not long because they were generated by single pass sequencing, with an average length of ~580 bp [13,17]. In addition, the number of BAC end sequences is still limited, approximately 60,000 representing 25,677 paired reads. It is apparent that additional sequences specific for the physical map contigs will greatly enhance the anchorage ability of such sequences. The objective of this study was to generate more sequence tags from distinct physical contigs, namely physical map contig-specific sequences, to allow vast majority of genome sequence contigs to be anchored to physical map contigs, at a reasonable cost. Here we report a simple strategy for the production of physical map contig-specific sequences, their assembly, and their anchorage capability of random genome sequence contigs to the physical map contigs. 

## Results

### Strategy for generating physical map contig-specific sequences

**Figure 1:**
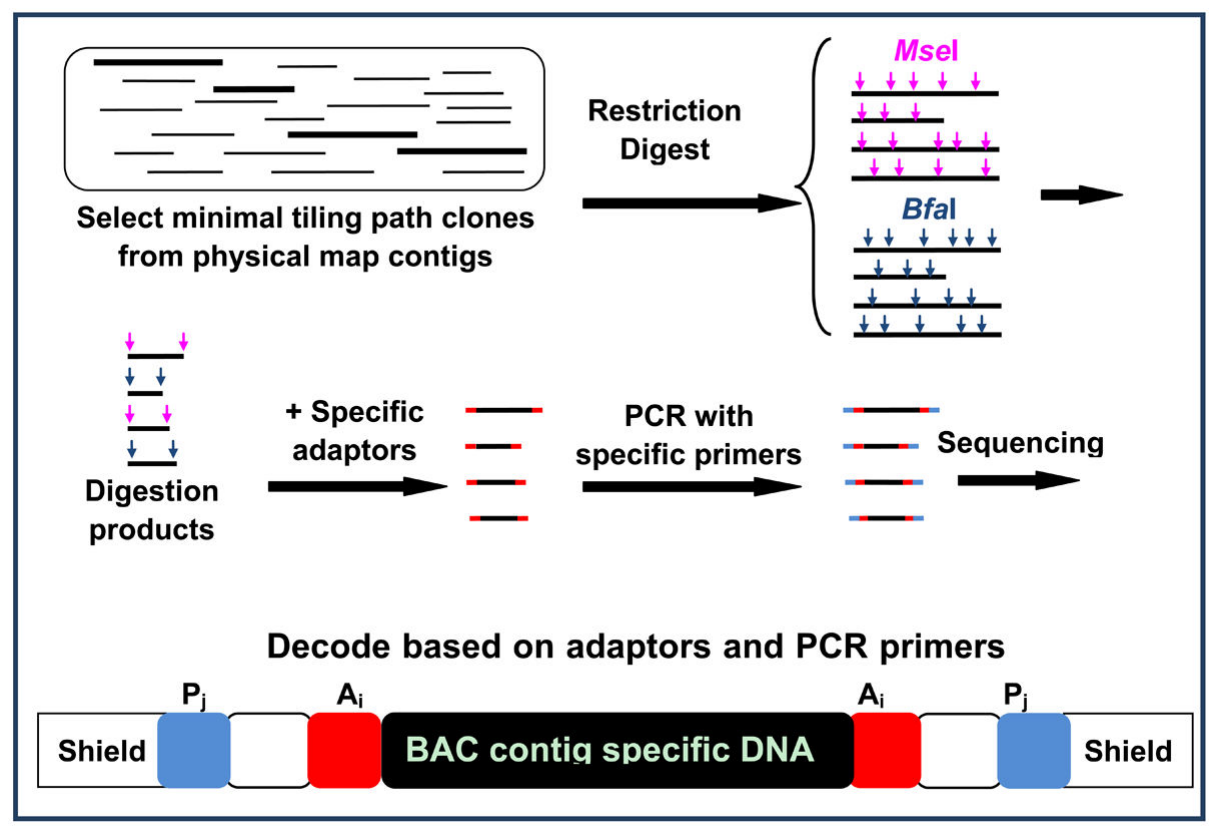
Flow chart illustrating the physical map contig-specific fragment preparation. The minimal tilling path BAC clones from each physical contig were selected (highlighted). The pooled BAC DNA from each physical map contig was digested with two 4-bp restriction enzymes, *Mse* I and *Bfa* I, respectively. The digestion product was then ligated with in-house designed adaptors, followed by PCR using in-house designed primers. The combination of adaptor and primer formed a specific tag representing each physical contig ID. All PCR products with a physical map contig-specific tag then were pooled together, and sequenced using Illumina HiSeq 2000 platform.

The strategy for generating physical map contig-specific sequences is illustrated in Figure 1. The strategy includes the following steps: 1) selection of clones representing a minimal tiling path (MTP) of each physical map contig; 2) Pooling of BAC DNA representing MTP of each contig; 3) Restriction digestion by using two 4-bp cutter restriction endonucleases, separately. The two restriction enzymes, *Mse* I and *Bfa* I, were used for the digestion. The basic requirements of the two restriction enzymes were: a) they have different recognition sequences such that their restriction fragments are overlapping, allowing sequences to be assembled; and b) their restriction fragments harbor compatible 5’-TA overhangs such that the restriction fragments can be ligated to the adaptor sequences with a 5’-TA overhang; 4) Ligation of specific bar-coded adaptors to fragments generated from each physical map contig, separately; 5) PCR amplification of the specific bar-coded fragments using barcoded PCR primers for fragments generated from each physical map contig; and 6) Illumina sequencing of the PCR-amplified fragments, followed by decoding of the sequences to specific physical map contigs using specific bar coding of the adaptors and the PCR primers (Figure 1).

### Barcoding Tags design

In this strategy, the physical map contig-specific sequences were designated by a two-dimensional tagging strategy using both the adaptor and the PCR primer to reduce the total number of bar codes required for the 1,824 physical map contigs. For instance, if only a single bar-code is used, the “labeling” of the 1824 physical map contigs would require 1,824 different bar codes. When the two-dimensional tagging strategy was used, 38 different adaptors and 48 different PCR primers were used (total of only 86 sequence tags), which allowed a combination of 38 x 48 bar codes, i.e., 1,824.

Each adaptor was designed to include a 5 base pair specific bar code immediately adjacent to the 5’-TA-overhang, proximal to the fragments for ligation, and a 13 base pair common sequence for PCR. Each PCR primer was designed to include 5 base of common sequence as the “shield” sequence, then 5 base of specific bar-coded sequence, followed by a 13 base sequence complementary to the 13 base common sequences of the adaptors. Thus, the adaptors were 18 bp long, and the PCR primers were 23 bases long (Table S1). 

All 1,824 pooled BAC DNAs were arrayed into two dimension 38(row) x 48(column) using 20 96-well plates, in which the row represented one sets of tags, A_i_ , where i = 1, 2, 3, …38; and the column represented another set of tags, P_j_, where j = 1, 2, 3, …48. As such, each pool of PCR products represents fragments derived from a single physical map contig with A_i_ and P_j_ at the ends. The adaptor and primer sequences are presented in Table S1.

### The selection of the minimal tiling path

Two physical maps were published [25,26]. One was constructed using the BAC library CHORI 212 [27] with 3,307 contigs, and the other was constructed using BAC library from a gynogenetic channel catfish [28] with 1,782 contigs [25]. We have merged the two physical maps together (unpublished) with 1,824 physical contigs that include the BAC clones from the CHORI 212 library. A total of 6,701 BAC clones representing the minimal tilling path from 1,824 physical map contigs were selected and cultured for BAC DNA preparation. On average, ~ 4 BAC clones were selected in each physical map contig.

### Generation of physical map contig-specific sequences

**Figure 2:**
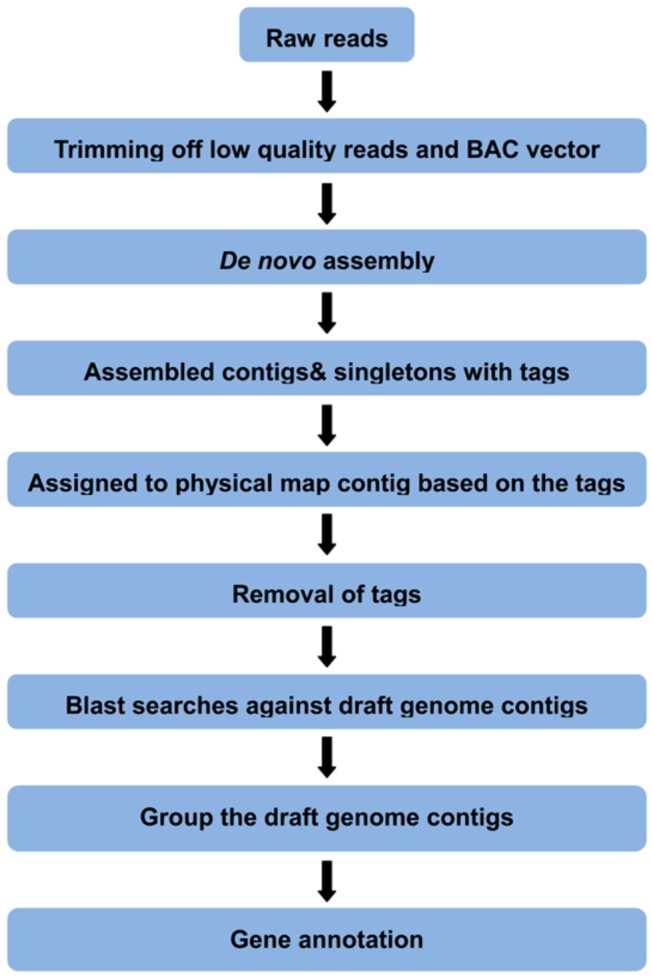
The workflow of data processing. The raw reads were first trimmed off the low quality reads (Q20) and BAC vectors. *De* *novo* assembly was then conducted with the filtered high quality reads. The assembled contigs with tag on it, plus singletons which have tag on it were then assigned to each physical contig, based on the specific tag. The tags were removed. The clean sequences were then used as queries to BLAST search against the draft catfish whole genome contigs. The targeted genome contigs were then retrieved and annotated.

**Figure 3:**
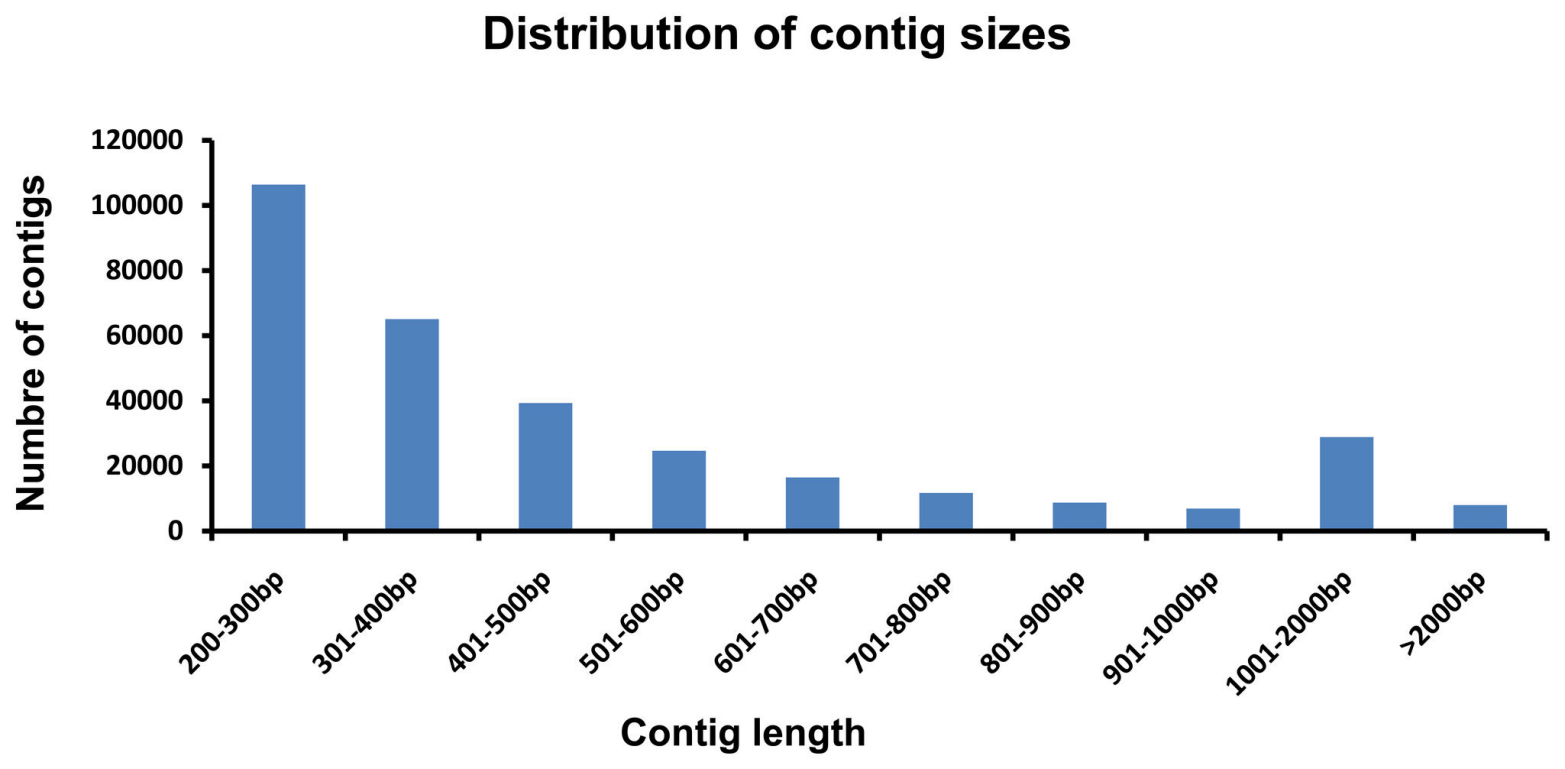
The distribution of contig sizes. The X axis represents the length of sequence contigs, starting with 200-300 bp, since the minimum length of the contig is 200 bp, followed by 301-400 bp, 401-500 bp, 501-600 bp, 601-700 bp, 701-800 bp, 801-900 bp, 901-1000 bp, 1001-2000 bp and > 2000 bp. The Y axis represents the number of sequence contigs.

**Figure 4:**
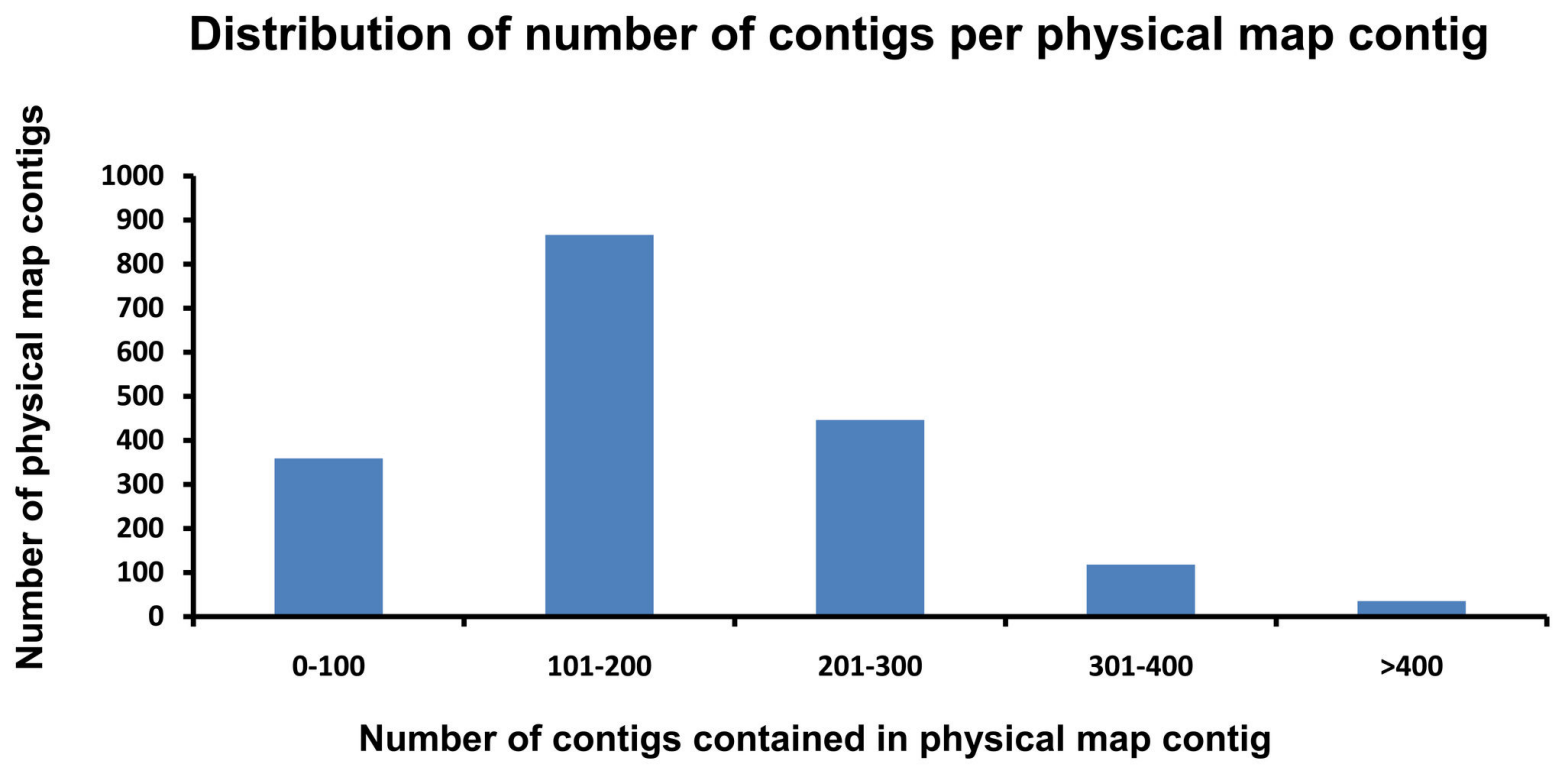
The distribution of number of contigs per physical map contig. The X axis represents the number of sequence contigs contained in each physical map contig, starting with 0-100, followed by 101-200, 201-300, 301-400 and > 400. The Y axis represents the number of physical map contigs.

Following the strategy described above, all PCR products were pooled together and sequenced using Illumina HiSeq2000. Figure 2 shows the overall processing with the raw data. A total of 334,381,996 100-bp paired-end reads were generated (Table 1). The sequence data have been deposited in NCBI Short Read Archive with the accession number SRR640312. Low quality reads (Q < 20) were filtered and reads shorter than 20-bp were discarded, with CLC Genomics Workbench 5.5. After trimming, there are a total of 328,229,917 high quality reads. Of the 328,229,917 reads, there are 94,111,841 reads attached with the physical map contig-specific tags. The high quality reads were used for *de novo* assembly, by using ABySS 1.3.0 with a k-value of 55. A total of 315,277 assembled contigs were generated after assembly, with average contig size of 552 bp, and N50 of 670 bp. The distribution of those assembled contig sizes is shown in Figure 3. Each physical map contig contained 173 assembled contig on average. As shown in Figure 4, the majority of physical map contigs contain around 200 assembled contigs with tags. Of all assembled contigs, 6,951 contigs (~ 4 contigs per physical map contig on average) can be mapped to multiple region of the catfish genome, highly likely due to the repetitive sequences in catfish genome. Considering the specificity, we excluded those contigs in the anchoring process in this study. Both contigs and 57,545,833 singletons were identified as the physical map contig-specific sequences, and assigned to each physical contig based on its attached specific tags (Table 1).

**Table 1:** Summary of the physical map contig-specific sequences.

Total number of raw reads	334,381,996
number of trimmed reads	328,229,917
Number of reads with tags	94,111,841
Number of assembled contigs	315,917
Number of assembled contigs with tags	315,277
Average number of assembled sequence contigs with tags per physical map contig	173
Number of singleton reads with tags	57,545,833
Total number of tagged sequence contigs &singletons	57,861,110
Total number of BAC end sequences available for the 1,824 physical map contigs	42,616
Number of BAC end sequences from the 1,824 physical map contigs with hits from tagged sequences	31,809

### Anchoring the draft genome sequence contigs to the physical map contigs

After the identification of physical map contig-specific sequences in each physical map contig, the barcoding tags were first trimmed as they were not part of the genome sequences from catfish. After trimming, the clean physical map contig-specific sequences represent the randomly distributed sequences in each physical contig, and serve as the anchor point to link the catfish draft genome sequence contigs (unpublished data) to the physical map contigs. 

In order to determine how many genome sequence contigs can be anchored to the physical map contigs by the newly generated physical map contig-specific sequences (PMCSS) (contigs plus singletons), BAC end sequences, with or without PMCSS were used to BLAST the catfish whole genome sequence contigs. As shown in Table 2, with just the existing BAC end sequences, 27,770 whole genome sequence contigs had significant hits to the BES. However, this number was drastically enhanced with the PMCSS, bringing the number of whole genome sequence contigs with significant hits to 156,457, i.e. 61% of whole genome sequence contigs had significant hits to the BES plus PMCSS, as compared to only 11% when only BES were used. In terms of genome sequence contig length, an even greater % of the whole genome sequence contigs were anchored by using the newly generated PMCSS. Only 26% of the whole genome sequence length was anchored by using BES only, and when the PMCSS were also used, over 79% of the whole genome sequence assembly were anchored (Table 2).

**Table 2:** Comparison of effect on comparative genomics study with and without physical map contig-specific sequences (PMCSS).

	**With BES only**	**With BES &PMCSS**
Number of unique genome sequence contig hits	27,770	156,457
% of genome contig hits	11%	61%
Total length of genome contig hits	202 Mb	608 Mb
% of total length genome contig hits	26%	79%
Number of unique gene hits	6,732	16,680
Total length of genome sequences anchored into chromosome-scale scaffolds	162 Mb	421 Mb
% of total length genome sequences anchored into chromosome-scale scaffolds	21%	55%
Number of unique gene anchored into chromosome-scale scaffolds	5,489	12,423

We also assessed the impact of PMCSS on scaffolding by examination of the genes that can be anchored to the whole genome sequence contigs. When only BES was used to anchor the whole genome sequence contigs, a total of 6,732 genes were identified within the whole genome sequence contigs with hits to BES. This number was drastically enhanced, to 16,680 genes (Table 2), when the PMCSS were also used, confirming strong anchorage capability of the PMCSS. 

We previously reported the integration of the catfish physical map with its linkage map by using BAC end sequences-derived microsatellites [23]. The total physical lengths of all the physical map contigs that were genetically mapped to the linkage groups (chromosomes) were approximately 162 Mb. By using also the PMCSS, a total of 421 Mb of genome sequences can now be anchored to linkage groups (chromosomes). With the total length of the draft genome assembled whole genome sequences being 766 Mb, the PMCSS allowed 55% of the physical genome to be anchored to chromosome-scale scaffolds. The total number of genes that could be anchored to linkage groups were increased from 5,489 genes to 12,423 genes when the PMCSS were used (Table 2). 

## Discussion

Assembly of whole genome sequences using next generation sequencing short reads is a great challenge. Such a challenge becomes even a greater challenge for teleost fish genomes because the presence of highly abundant interspersed repetitive elements such as Tc1 transposons, and long tracts of simple sequence repeats. As a result, whole genome sequence assemblies reflect the status of repetitive elements with contigs broken at the repetitive sequences, resulting in two unwanted consequences: 1) the segmented genome assembly with a large number of contigs; and 2) the repetitive elements stay unassembled such that the completeness of the genome assembly is reduced. However, for many fish species, the most significant issue for practical applications is the ability to scaffold the relatively short genome sequence contigs to large, chromosome-scale scaffolds. We have previously generated genome resources for scaffolding such as BAC end sequences [13,17]. However, these BAC end sequences were able to only scaffold approximately 21% of the genome to chromosome-scale scaffolds. Additional resources for scaffolding are greatly needed. This project intended to generate sequences specific to physical map contigs to provide greater scaffolding capacity. 

Here we report a simple strategy for the generation of such physical map contig-specific sequences using a two-dimensional tagging strategy. This strategy is very simple. It is based on a two-dimensional specific sequence bar coding, one bar code on the adaptors linked to each physical map contig fragments, and the other linked to PCR primers used to amplify fragments from each physical map contigs. This two dimensional design allowed the reduction of total number of bar codes. For instance, to differentiate the 1,824 physical map contigs, a total of at least 1,824 bar codes are needed. Synthesis of 1,824 primers would cost much more. By using the two dimensional design, a total of 86 primers were used, significantly reducing the cost without any loss of the differentiating power. 

The most tedious step is the DNA preparation, adaptor ligation and PCR amplification step for each of the physical map contigs because each physical map contig is treated as a separate sample. In our case, this involves 1,824 physical map contigs, thus 1,824 separate samples for DNA isolation, adaptor ligation, and PCR amplification. Considering how much information this project provide, such a tedious step is still worthwhile. A total of over 330 million reads were obtained, with over 28.7% harboring the sequence tags specific to each of the physical map contigs, thus allowing generation of 94,111,841 tagged sequences. The shearing of the PCR products during sequencing process weakens the barcoding efficiency since the barcodes are attached only at the ends of the PCR products. To strengthen the barcoding ability, two 4-bp cutters that had the same 5’-overhang but different recognition sequences were used in this study. This would allow overlapping fragments to be generated such that the sequences can be assembled. As shown in Table 1, a total of 315,917 contigs were generated after assembly, of which 315,277 (99.8%) assembled contigs harbored the physical map contig-specific tags. The ability to assemble these sequences could also allow longer and greater percentage of sequences to be assigned to physical map contigs (Table 1). 

We made the assessment of how these physical map contig-specific sequences affected the scaffolding capabilities. Several parameters were used including the number of whole genome sequence contigs that can be anchored to the physical map, the physical lengths of the whole genome sequence contigs that can be anchored to the physical map, and the number of genes that can be anchored to the physical map. With all these indicators, the physical map contig-specific sequences provided a high ability of enhancing the scaffolding capability (Table 2). 

However, we realize that with just the physical map contig-specific sequences generated here, the sequences are anchored into 1,824 “pools” of sequences belonging to the physical map contigs. We cannot yet resolve the sequence stacking without additional information such as scaffolding using mate-paired reads of various sizes. Apparently, the ability to generate larger sequence contigs and the ability to place sequence contigs into linear scaffolds using mate-paired reads with various insert lengths are all very important for the whole genome sequence assembly, and such genome resources are being generated. Nonetheless, these physical map contig-specific sequences will provide another level of scaffolding capability for the whole genome assembly and annotation of the catfish genome. 

In the absence of a well-assembled whole genome sequence assembly, the physical map contig-specific sequences can greatly enhance the comparative genomics studies. For instance, most genes located within a physical map contig can now be readily identified. All anchored genome sequence contigs within a physical map contig can be used to search against Uniprot database, generating the genes that are located within the physical map contig [29]. Using all the annotated genes in all the physical map contigs, along with map integration information [23], will greatly benefit the genome-wide comparative analysis of catfish. As shown in Table 2, if using BES in each physical contig alone as the anchor point to link genome contigs and physical contigs, there are 27,770 genome contigs anchored, resulting in 6,732 unique genes annotated, of which 5,489 genes can be anchored to the chromosome-scale scaffolds in linkage map. Addition of the physical map contig-specific sequences as anchor points, the number of anchored genome contigs increased to 156,457, resulting in 16,680 unique genes annotated, of which 12,423 genes can be anchored to the chromosome-scale scaffolds. Consequently, this would greatly improve the genome-wide comparative analysis of catfish. However, as discussed above, sequence stacking, which would also leads to gene stacking, has yet to be resolved with additional genome resources before a thorough whole genome comparative map can be constructed for catfish, but a forest view of the whole genome, even with sequence stacking, still provide useful genome information.

## Conclusions

In this study, we developed a strategy to generate physical map contig-specific sequences in an economical way, by using a two-dimensional tagging strategy. Analysis of the physical map contig-specific sequences indicated that such sequences can add significant scaffolding capabilities, serving as a useful resource for whole genome sequence assembly and comparative genomic studies. However, such physical map contig-specific sequences cannot yet resolve sequence stackings, but rather group random genome sequence contigs into physical map contigs. 

## Materials and Methods

### BAC clone culture and BAC DNA isolation

The catfish BAC clones from a minimum tiling path covering each physical contig from the CHORI-212 BAC library [27] were selected. The BAC DNA isolation was conducted as previously described [13], with modifications. Briefly, BAC clones were transferred from 384-well plates to a 96-well culture block, which contained 1.5 ml of 2X YT medium with 12.5 µg/ml chloramphenicol and grown at 37°C overnight at 300 rpm. The block was centrifuged at 2000 x *g* for 10 min in an Eppendorf 5804R bench top centrifuge to collect bacteria. The culture supernatant was decanted and the block was inverted and tapped gently on paper towels to remove remaining liquid. BAC DNA was isolated using the Perfectprep™ BAC 96 kit (Eppendorf North America, Westbury, NY) according to the manufacturer’s specifications.

### Physical map contig-specific tag design

All 1,824 physical contigs were arranged into two-dimension 38(row) x 48(column) table. Instead of designing 1,824 tags for each physical contig, 38 adaptors, A_i (i=1,2,…..38),_ and 48 primers, P_j (j=1,2,…48)_ with distinct barcodes, were designed. A 5-bp distinct barcode was designed for each adaptor and primer. The combination of A_i_ and P_j_ served as a distinct tag for each physical contig. The detailed sequences of primers and adaptors are shown in Table S1.

### Sample preparation and sequencing

An amount of 100 ng BAC DNA from each physical contig was digested with 4-bp restriction enzymes *Mse* I and *Bfa* I, respectively. The two enzymes recognize different 4-bp sites but produce same 5’-TA overhangs. The digestion was conducted following the manufacturer’s specifications, with modifications. Briefly, 100 ng BAC DNA was digested using 1 U of *Mse* I / *Bfa* I in a final volume of 5 µl reaction. The digestion mixture was incubated at 37 °C for 3 hours, and then inactivated at 80 °C for 20 minutes. After digestion, 0.5 µl [5 µM] in-house-designed adaptor was added for ligation using T4 ligase at 4 °C overnight. 1 U T4 ligase was used for each reaction in a final volume of 10 µl.

One microliter ligation product was used as template for amplification with the following reaction cocktail: 1 µl 10X buffer, 1 U polymerase, 0.4 µl MgCl_2_ [50 mM], 0.8 µl dNTPs [2.5 mM], 2µl [5 µM] in-house-designed primers. Reactions were conducted in a thermocycler with the following thermal profile: 72 °C for 2 minutes, denaturing at 94 °C for 3 minutes, followed by 30 cycles of 94 °C for 30 seconds, 58 °C for 1 minutes and 72 °C for 3 minutes. A final extension was performed at 72 °C for 15 minutes to complete the PCR. Every single reaction was checked by electrophoresis on a 1% agarose gel and documented with a gel documentation system (Bio-Rad, Hercules, CA). All PCR products were pooled together and purified using the Qiaquick PCR Purification kit (Qiagen). A total of 1 µg high quality PCR products were sent to Genomic Services Lab at HudsonAlpha Institute for Biotechnology (Huntsville, AL) for sequencing using Illumina HiSeq 2000.

### Identification of physical map contig-specific tags

CLC Genomics Workbench 5.5 (CLC Bio, Cambridge, MA) was used to remove the BAC vector and low quality reads, with quality score limit of 0.01 (Q20). Illumina 100-bp PE reads shorter than 20bp were discarded. *De novo* assembly was conducted by using ABySS 1.3.0, with a k-mer value of 55. A script was used to search the physical map contig-specific tags in all Illumina reads as well as the assembled contigs. After physical map contig-specific sequence identification and assignment to each physical contig, the specific tags were then trimmed using CLC Genomics Workbench 5.5.

### Identification of anchored genome contigs

The clean physical map contig-specific sequences were used as queries to search against the draft catfish genome sequences by BLASTN, with an E-value cutoff of 1e-20. The query sequences with multiple hits of genome contigs were considered non-specific and discarded. Only the query sequences with a single hits and identity value greater than 98% were considered as specific sequences. The corresponding genome contig hits were retrieved to use as queries to BLASTX search against the Uniprot database for gene annotation, with an E-value cutoff of 1e-10. 

## Supporting Information

Table S1 **The sequences of adaptors (Ai, i=1-38) and primers (Pj, j=1-48).**
(XLSX)
